# What Does Androgen Receptor Signaling Pathway in Sertoli Cells During Normal Spermatogenesis Tell Us?

**DOI:** 10.3389/fendo.2022.838858

**Published:** 2022-02-24

**Authors:** Jia-Ming Wang, Zhen-Fang Li, Wan-Xi Yang

**Affiliations:** The Sperm Laboratory, College of Life Sciences, Zhejiang University, Hangzhou, China

**Keywords:** androgen receptor, Sertoli cell, signaling pathway, spermatogenesis, androgen insensitivity syndrome

## Abstract

Androgen receptor signaling pathway is necessary to complete spermatogenesis in testes. Difference between androgen binding location in Sertoli cell classifies androgen receptor signaling pathway into classical signaling pathway and non-classical signaling pathway. As the only somatic cell type in seminiferous tubule, Sertoli cells are under androgen receptor signaling pathway regulation *via* androgen receptor located in cytoplasm and plasma membrane. Androgen receptor signaling pathway is able to regulate biological processes in Sertoli cells as well as germ cells surrounded between Sertoli cells. Our review will summarize the major discoveries of androgen receptor signaling pathway in Sertoli cells and the paracrine action on germ cells. Androgen receptor signaling pathway regulates Sertoli cell proliferation and maturation, as well as maintain the integrity of blood-testis barrier formed between Sertoli cells. Also, Spermatogonia stem cells achieve a balance between self-renewal and differentiation under androgen receptor signaling regulation. Meiotic and post-meiotic processes including Sertoli cell - Spermatid attachment and Spermatid development are guaranteed by androgen receptor signaling until the final sperm release. This review also includes one disease related to androgen receptor signaling dysfunction named as androgen insensitivity syndrome. As a step further ahead, this review may be conducive to develop therapies which can cure impaired androgen receptor signaling in Sertoli cells.

## 1 Introduction

Male infertility is currently a major problem worldwide and causes substantial psychological and social distress (1). It not only puts a huge economic burden on patients, but also poses a great challenge to the health-care system. Male infertility is due to abnormal sperm parameters in the male partner and contributes to 50% of all cases of infertility, highlighting the importance of normal spermatogenesis (2, 3).

Spermatogenesis is a complex process that is under precise regulation. Starting from spermatogonia stem cell (SSC) producing differentiated spermatogonia, spermatogonia transform into spermatocytes which then undergo meiotic divisions to produce round spermatids (4). Round spermatids undergo cytodifferentiation to form spermatozoa, which are ultimately released to the lumen (5).

As the only somatic cell type in seminiferous tubules (6), Sertoli cells (SCs) make normal spermatogenesis possible by providing the nutrition necessary for the development of germ cells (7), forming blood-testis barrier (BTB) between SCs in mammalian testes (8, 9), attaching to germ cells (GCs) through adherens junctions (10) and phagocytosing apoptotic GCs for recycling (11). SCs function like a nurse to take good care of spermatogenesis (12). These functions of SCs are regulated by both extrinsic and intrinsic factors. The former factors include hormone and paracrine molecules and the latter include genomic regulators. Here, we review androgen receptor (AR) signaling pathway in SCs, a pathway that provides an excellent example of a combination of extrinsic and intrinsic regulation. AR begins its expression 3-5 days after birth in rodent testis while AR expression begins about 5 months after birth in men (13, 14). In men, its expression in SCs peaks during stage III of the six stages (15). AR signaling pathway can be classified into classical signaling pathway and non-classical signaling pathway with different functions and different efficiencies. It has been shown that abnormal AR signaling can impair spermatogenesis (16). Although decades of studies have brought us a better understanding of AR signaling in spermatogenesis using altered testosterone signaling model, Sertoli cell androgen receptor knockout model, specificity-affecting androgen receptor knock in model as well as Ribo-Tag mouse model, not enough information has been gained to lift the veil of its beauty. As a result, it is necessary to review current work about androgen receptor signaling pathway in SCs and put forward suggestions for future studies. Ultimately, all of our hard work aims to achieve “bench to bedside” translation that can be used to develop therapies for illnesses related to hormone dysfunction.

**Figure 1:**
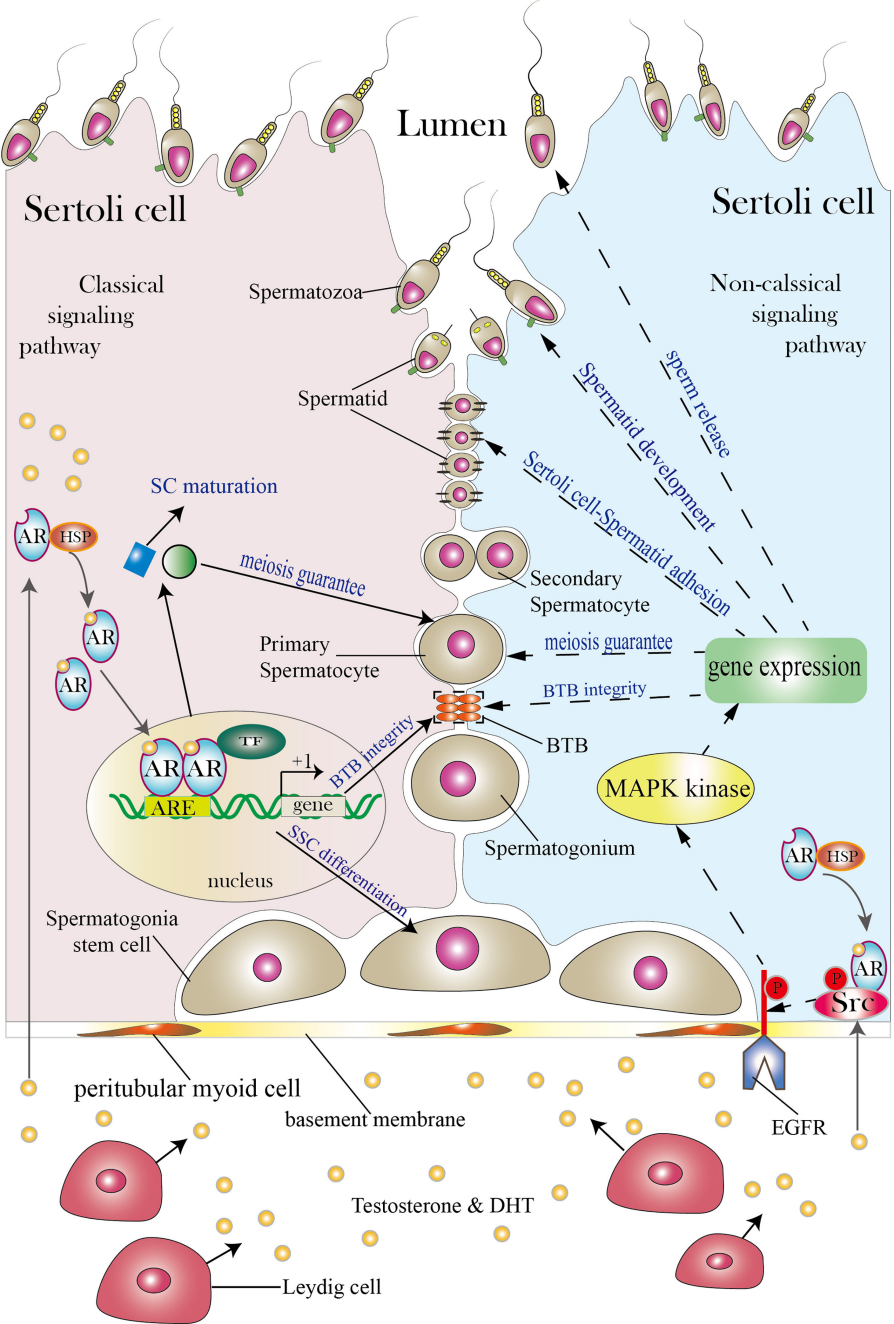
Classical and non-classical AR signaling pathways in SCs. The SC shown on the left illustrates the classical signaling pathway, while the SC shown on the right illustrates the non-classical signaling pathway (both classical and non-classical signaling pathways can exist in a single SC; the two pathways are presented in separate cells for simplicity). Between the two neighboring SCs are germ cells (spermatogonia stem cells, spermatogonia, primary spermatocytes, secondary spermatocytes, spermatids and spermatozoa). SCs function as a nurse to take good care of normal spermatogenesis as spermatogonia stem cells develop into spermatozoa in the lumen. Androgen secreted by Leydig cells diffuses through the plasma membrane and binds to the AR complex in the cytoplasm to activate the classical signaling pathway. The AR then detaches from the HSP complex and enters the nucleus to form AR dimers. Dimerized ARs bind to the AREs of their target genes and thereby regulate their transcription. The classical signaling pathway can promote SC maturation, induce SSC differentiation, guarantee spermatocyte meiosis, and ensure BTB integrity. The non-classical signaling pathway begins with translocation of the AR from the cytoplasm to the plasma membrane. There, the AR interacts with Src and causes it to phosphorylate EGFR, and this in turn activates MAPK kinases to regulate targeted gene transcription. The non-classical signaling pathway can regulate processes including BTB integrity, spermatocyte meiosis, Sertoli cell-Spermatid adhesion, spermatid development and sperm release. The three orange ovals in the figure represent the BTB, and the two black lines represent cell–cell adhesion. TF is short for transcription factor. Areas for which controversy exists are not included in the figure but are discussed in the article.

We surveyed articles in the PubMed database using the following search terms: androgen receptor*, Sertoli cell*, spermatogonia*, maturation and differentiation*, spermatogenesis*, meiosis*, spermatid* and androgen-insensitive syndrome*. We will present this review at the cellular/molecular level in six parts: SC proliferation and maturation, SSC self-renewal and differentiation, spermatocyte meiosis, BTB integrity maintenance, Sertoli cell - Spermatid adhesion, and sperm release (**Figure 1**). The experimental models involved in this review include mice, rats, boars, lambs, zebrafish and humans. We include some contradictory results in this review and present our opinions. We also discuss androgen insensitive syndrome (AIS), briefly reviewing its pathogenesis, diagnosis and offering suggestions for better diagnosis and treatment.

## 2 Structure of Androgen Receptor

The gene encoding AR is located on the X chromosome, belonging to nuclear receptor subfamily 3, group C, member 4. It consists of 8 exons that encode 4 domains: an N-terminal domain (exon 1), a DNA-binding domain (exon 2 and exon 3), a hinge region that contains the nuclear localization signal (exon 3 and exon 4) and a ligand-binding domain (exons 4-8) (17, 18).

The N-terminal domain contains activation function domain-1 which is constitutively active (15). The DNA-binding domain contains two zinc fingers to complex with its hormone response element, where each of them is coordinated by four cysteines. One zinc finger is involved in direct DNA binding mediated by the P(proximal) box, which recognizes the specific hormone response element half-site 5′-AGAACA-3′. The other zinc finger is involved in a “head-to-head” receptor dimerization through the D(istal) box (19–22). The hinge region contains the nuclear localization signal which is essential for nuclear import. Residues from the major nuclear localization signal site 629-RKLKKL-634 contribute to importinα binding (23–25). Then the ligand-binding domain consists of 11 α-helices and two small, two-stranded β-sheets arranged in a typical three-layer antiparallel helical sandwich fold (26, 27). Binding of androgen to ligand-binding domain can activate the receptor. Both of these structures are important for androgen receptor function, though the structure of full-length androgen receptor has not been solved yet.

## 3 Overview of Androgen Receptor Signaling Pathway in Sertoli Cells

In adult rodent testes, the AR expression level in SCs is low except during stage VI-VIII of the seminiferous epithelium (28). In men, its expression in SCs peaks during stage III of the six stages (29). This stage-specific peak of AR expression in SCs is coordinated with initiation of testosterone-dependent processes that is essential for spermatogenesis such as appearance of preleptotene spermatocytes and the initiation of meiosis during stages VII–VIII, as confirmed by Sertoli cell androgen receptor knock out model and androgen receptor knock out model (5). It is reasonable that AR signaling in SCs mediates paracrine action on germ cells and autocrine action on themselves to form a microenvironment for meiosis completion.

Stimulated by luteinizing hormone in the pituitary gland which is part of the hypothalamus-pituitary-gonadal axis, Leydig cells produce androgen. When androgen saturates the AR of SCs and binds to AR, the AR signaling pathway is activated (30). Testosterone is the most abundant androgen produced in the testis. Another important androgen is dihydrotestosterone (DHT), which is produced by peripheral tissues where 5α-reductases reduce testosterone to DHT (31, 32). When hormone levels in the targeted tissue are low, DHT is more potent than testosterone due to its higher binding affinity for AR. When the testosterone level in the testis is high, there are no differences between testosterone and DHT (33). Testosterone concentrations in the testes of men (340–2000 nM) are 25 to 125-fold greater than those in serum (8.75–35 mM), similar to the situation in rodent testes (5). The testosterone level needed to maintain spermatogenesis is approximately 10-25% of the intratesticular testosterone level (34). AR signaling pathway in SCs can be divided into classical signaling pathway and non-classical signaling pathway. The main differences between them lie in androgen binding location and response speed. Androgen binds to cytoplasmic AR in classical signaling pathway while in non-classical signaling pathway androgen binds to AR on plasma membrane. Compared with non-classical pathway, classical pathway requires more time to show transcriptional activity change after androgen binding to androgen receptor (35).

### 3.1 Classical Signaling Pathway

Before binding to androgen, AR in the cytoplasm binds to chaperones and cochaperones, such as heat shock proteins (HSP) HSP23, HSP40, HSP56, HSP70, HSP90 *via* its ligand-binding domain (17). When androgen in the cytoplasm binds to AR, AR detaches from chaperone and cochaperone protein complexes and exposes its ligand-binding domain (23, 24, 36). With the help of importins, the AR monomer transits into the nucleus, where 2 AR monomers dimerize to create AR homodimers. AR homodimers then bind to androgen response elements (AREs) in the promoter regions of targeted genes to regulate their transcription (37, 38). In addition, activation function domain-1 presenting in AR dimer N-terminal domain and activation function domain-2 presenting in its ligand-binding domain can recruit coactivators and corepressors that activate or repress targeted gene transcription activity (17). However, the binding sites for AR homodimers are not restricted to AREs; AR homodimers can also bind to some response elements for other transcription factor (39, 40). Notably, the classical AR signaling is relatively slow, usually 30-45 minutes is needed to induce activation or suppression of transcription, not considering the time required for protein synthesis and secretion (35).

### 3.2 Non-Classical Signaling Pathway

To date, three types of non-classical signaling pathway have been identified in the testis. Here is the first type, as well as the main type. When the intratesticular testosterone level is relatively low (10-250 nM), testosterone binds to the AR located in the cell membrane and activates the non-classical AR signaling pathway within 1 minute (41). In the TM4 Sertoli cell line, the transport of cytoplasmic ARs to the membrane is facilitated by caveolin-1 (42). Testosterone binds to membrane AR which then interacts with the SH3 domain of SRC proto-oncogene (Src). Src then phosphorylates the epidermal growth factor receptor (EGFR) which activates Ras kinase. This activates MAPK cascades (Ras-Raf-MEK-ERK). Phosphorylated ERK can phosphorylate ribosomal protein S6 kinase A1(p90^Rsk^). Activated p90^Rsk^ translocate into nucleus, where it activates transcription factors such as cAMP-response element-binding protein (CREB), which binds to the cAMP-response element (CRE) of the targeted gene to regulate transcription (41, 43).

An *in vitro* study in which the TM4 cell line was exposed to testosterone levels of 10-100 nM demonstrated that testosterone can also activate a second non-classical signaling pathway, the phosphatidylinositol 3 kinase (PI3K)/Akt pathway, directly by activating the PI3K subunit p85α. Phosphorylated Akt can activate Src and this facilitates the translocation of cytoplasmic AR to the plasma membrane (44). In addition to these *in vitro* studies, some *in vivo* studies also showed that testosterone can activate the non-classical signaling pathway in SCs (35, 45).

Another type of non-classical signaling pathway has only been found in immature Sertoli cells. Testosterone causes depolarization of Sertoli cells within thirty seconds due to closed K^+^_ATP_ channel. This close is caused by G protein-mediated activation of phospholipase C. This action results in a rapid influx of Ca^2+^ and the activation of signaling molecules (46–48).

Recently, Zrt- and Irt-like protein 9 (ZIP9), a novel membrane-bound androgen receptor unrelated to classic AR, was found in the 93RS2 Sertoli cell line which has no cytoplasmic AR. ZIP9 participates in the phosphorylation of CREB and ATF1 *via* ERK1/2 to induce the expression of claudin proteins that are components of BTB. The relationship between AR signaling and ZIP9 signaling remains to be researched in the future (49).

## 4 Androgen Receptor Signaling Pathway in Sertoli Cells Can Regulate Spermatogenesis

### 4.1 Role of AR Mediated Signaling in Sertoli Cell Proliferation and Maturation

**Figure 2:**
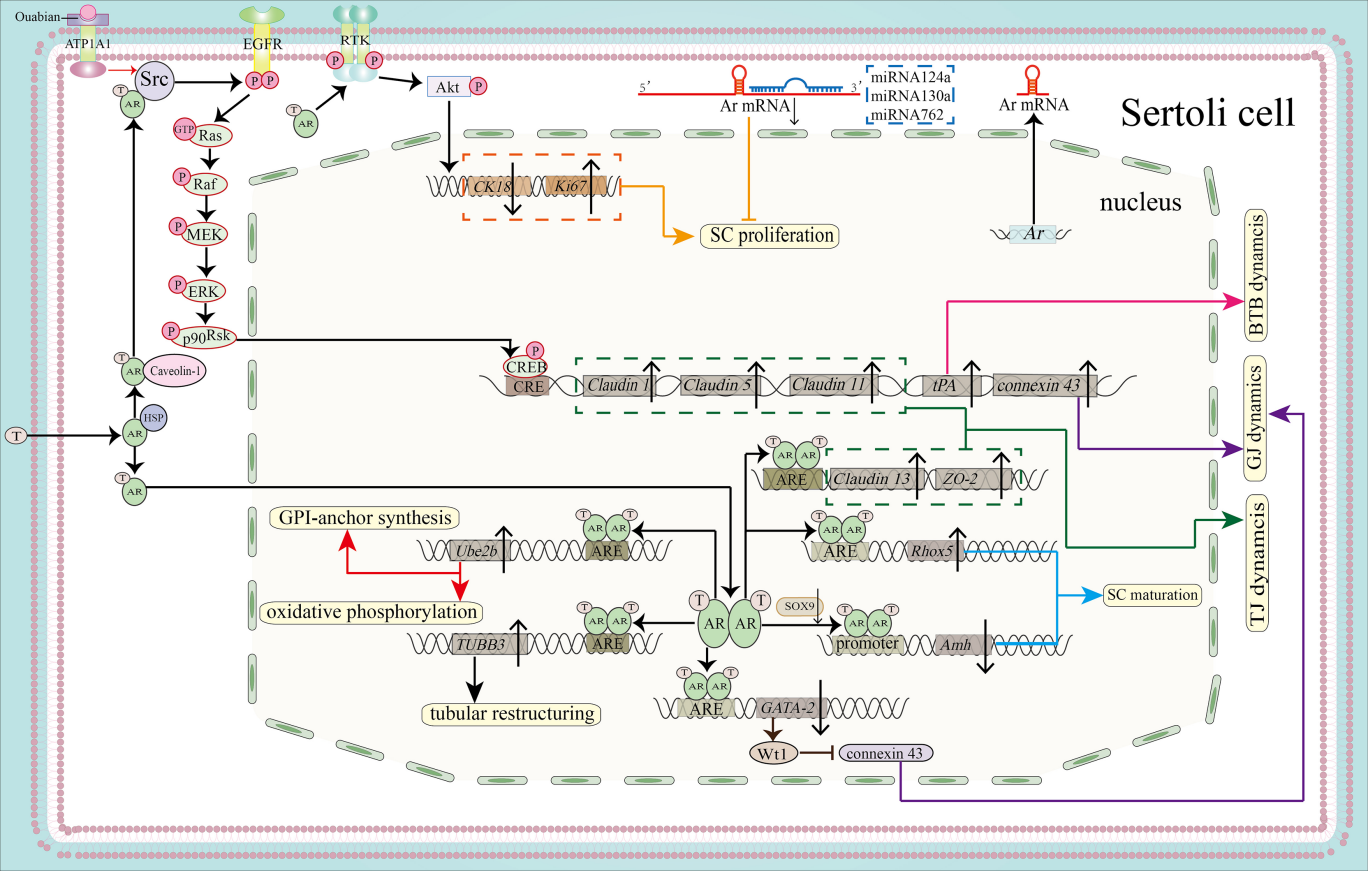
Function of the AR signaling pathway in SC proliferation, maturation and BTB integrity. By activating the PI3K/Akt pathway, the AR can promote SC proliferation by downregulating *CK18* and upregulating *Ki67* (shown in orange). MicroRNAs, including miR124a, miRNA130a and miRNA762, can inhibit SC proliferation by binding to the 3’-UTR of *Ar* mRNA (also shown in orange). AR dimers can bind to the AREs of *Rhox5* and *Amh* to promote the expression of *Rhox5* and inhibit the expression of *Amh*, leading to SC maturation (blue). Binding to the ARE of *TUBB3* induces tubular restructuring and achieve proper SC nuclei location, another process that is important in SC maturation. *Ube2b*, activated by the classical signaling pathway, is able to induce GPI-anchor synthesis and oxidative phosphorylation (shown in red). Both the classical and the non-classical signaling pathways are necessary for BTB integrity. The expression of *Claudin13* and *tight junction protein 2 isoform 3 (ZO-2)*is upregulated by classical AR signaling, together with upregulated expression of *Claudin1*, *Claudin5*, *Claudin11* and *tPA* by non-classical signaling. These molecules regulate BTB dynamics (shown in pink) and TJ dynamics (shown in green). In addition, AR dimerization binds to the promoter of *GATA-2* to inhibit its expression. GATA-2 can induce *Wt1* expression, while Wt1 inhibits the protein expression level of connexin 43 to regulate GJ dynamics. Ouabain increases the expression of *connexin 43 via* the ATP1A1/Src/EGFR/ERK/CREB signaling pathway (shown in purple).

SC proliferation occurs during fetal and early neonatal life in rodents, and in the fetal and peripubertal periods in higher primates when SCs undergo mitosis to increase their number (50). During the end of neonatal period or prepubertal period, SC proliferation stops. Then SCs maturation begins during puberty period, switching from an immature, proliferate state to a mature, non-proliferate state during which SCs establish BTB and acquire the ability to sustain spermatogenesis (51) AR signaling can participate in SC maturation process. Although it remains to be investigated if AR signaling in SCs influences SC proliferation, we still review studies in this field (**Figure 2**).

#### 4.1.1 Sertoli Cell Proliferation

According to our knowledge, final testis size, number of germ cells and sperm output are closely connected to the number of SCs. This emphasizes the significance of SC proliferation. When porcine testis was treated with AR antagonists during postnatal proliferation, an increased number of SCs per testis was detected (52). However, SC numbers were increased in young ram lambs following prenatal exposure to testosterone (53). This is not contradictory, because administration of exogenous testosterone increases negative hypothalamic feedback and may reduce intratesticular testosterone levels, leading to decreased androgen signaling. However, when comparing the number of SCs per testis in Sertoli cell androgen receptor knockout (SCARKO) mice and androgen receptor knockout (ARKO) mice with the control group, researchers found that the SCARKO mice did not show significant differences compared to the controls, whereas the ARKO mice showed a significant decrease in SC number, indicating that in mice AR in SCs has little impact on SCs proliferation (54, 55). A similar phenomenon was found when researchers used a testicular feminization mouse (*Tfm*) model that lacks functional AR in all cells (56). It is difficult to reconcile these results. We think that the expression of AR in SCs is undetectable or very weak during most of the periods in which these cells proliferate. While AR on other cell types such as peritubular myoid cells, which express AR at relatively high level, may contribute to SC proliferation. Hu et al. recently found that AR could interact directly with PI3K regulatory subunit p85α to activate kinase Akt and that this represses the expression of Cytokeratin 18 (CK18) (a marker of immature and dysfunctional SCs) and induces the expression of Ki67 (a marker of SC proliferation), thereby preserving SC proliferation in mice (57). Since the experiments were conducted on mature mice, additional experiments on prepubertal mice are needed to test this result, although it is true that some immature SCs are present in mature mice. Considering these results as a whole, it seems that role of AR signaling in SC proliferation is species-specific and phase-specific. We suggest that future studies test different species with the aims of understanding and combining their AR expression patterns in SCs, their intratesticular androgen levels and their SC proliferation patterns to provide a better understanding of the role of AR in SC proliferation.

#### 4.1.2 Sertoli Cell Maturation

Followed by cessation of SC proliferation comes to SC maturation. Around SC maturation, AR expression level is progressively upregulated until all SCs express AR (58). AR in SCs is key mediator for SC molecular and cytoskeleton maturation (59–61).

In molecular level, several molecules important for maturation are under AR signaling regulation (51) and the level of heterochromatin (62). Among them, Anti Mullerian hormone (AMH) has been widely researched. AMH is crucial for fetal sex differentiation by regressing the Müllerian ducts in male (13, 63). Secreted by immature SCs, AMH has become a biomarker for testicular function in males during the prepubertal period which is tightly regulated by AR (64). During the postnatal period, AMH level can be maintained by androgen epi-testosterone through non-classical AR signaling pathway when intracellular AR level is very low (65). Other transcription factors, such as sex determining region Y-box 9 (SOX9), steroidogenic factor 1 (SF1) can bind to their own response elements to promote *Amh* transcription (66). Around the onset of puberty, upregulation of AR expression downregulates AMH expression by blocking SF1 binding to its response elements or interacting with SF1 response elements to prevent SF1from exerting a stimulatory effect on *Amh* transcription (67). An *in vitro* study using TM4 Sertoli cells and C3H10T1/2 cell line demonstrated that AR could downregulate AMH expression by repressing SOX9, consistent with the findings in the azoospermia patients and mice *in vivo* (68).

Besides molecular level, AR signaling is necessary for SC cytoskeleton maturation. In SCARKO mice, relatively normal SCs nuclei were displaced from the basal basement and dispersed throughout the seminiferous tubules, probably resulting from the disrupted position of vimentin and the presence of thicker basal lamina, both of which participate in the linkage between nuclei and the cell membrane (69). Another group observed that SC nuclei formed one or two layered rings located in the center of tubule in mice in which AR function had been ablated (70). Suppressed expression of connexin 43(Cx43) in mice resulted in an intermediate state of SCs between proliferation and maturation, along with weaker AR immunostaining (71). The impaired SCs maturation observed in this model probably resulted from disrupted AR function. AR can also regulate microtubular composition and structure in SC so as to yield proper SC shape *via* Class III β tubulin (TUBB3) in mice and rats through classical signaling pathway (72).

Premature expression of ARs in postnatal mouse SCs resulted in fewer SCs, although GCs development accelerated in a gain-of-function mouse model (73). When the AR transgene was injected into a prenatal mouse model, precocious maturation of SCs occurred and limited the window for SC proliferation. The production of fewer SCs can lead to lower-than-normal sperm output (74).

#### 4.1.3 Factors Regulating Androgen Receptor Mediated Sertoli Cell Proliferation and Maturation

Precise regulation of Smad2/Smad3 is important both for SC proliferation and SC maturation (72). Type II A activin promotes immature testis growth along with timely appropriate AR expression *via* Smad3. The expression of Smad3 is then downregulated, and this is associated with cessation of SC proliferation and progression of the cells to terminal differentiation. AR activation can induce *Smad2* transcription, which will further promote SC maturation through shifting from using Smad3 only to using both Smad2 and Smad3 under activin A activation. In Smad3^-/-^ mice, delayed maturation of SCs was accompanied by reduced levels of AR and Smad2 (75).

In recent years, microRNAs (miRNAs) have been found to regulate SC proliferation and maturation. miR-124a binds to the 3’-UTR of *Ar* mRNA and downregulate AR expression, thereby inhibiting the proliferation of immature porcine SCs, while the coactivator really interesting new gene (RING) finger protein 4 (RNF4) binds to AR and promotes porcine SCs proliferation by elevating Proliferation cell nuclear antigen (a marker of cell proliferation) mRNA transcript levels (76). miR-762 promotes porcine SC maturation by inhibiting binding of RNF4 to AR (77). Moreover, miR-130a has been shown to inhibit AR expression, although its impact on SC proliferation and maturation remains to be elucidated (78). Additionally, the relationship of miR-133b (79) and miR-638 (80), which regulate SC proliferation and maturation, to AR remains to be investigated (**Figure 2**).

Importantly, the relationship between SC maturation and spermatogenesis is not well understood. We hypothesize that AR-regulated lipid metabolism in mature SCs can provide energy for spermatogenesis (81); for example, AR signaling in SCs induces the expression of ubiquitin-conjugating enzyme E2B (*Ube2b*) which regulates the expression of genes related to glycosylphosphatidylinositol (GPI)-anchor biosynthesis and oxidative phosphorylation (82). So does AR-regulated glucose uptake. The non-classical pathway participates in the regulation of glucose uptake *via* the cytochrome C oxidase (83). Also, BTB formed between SCs can facilitate normal spermatogenesis, as we will discuss later.

### 4.2 Role of AR Mediated Signaling in Spermatogonia Stem Cells Self-Renewal and Differentiation

**Figure 3:**
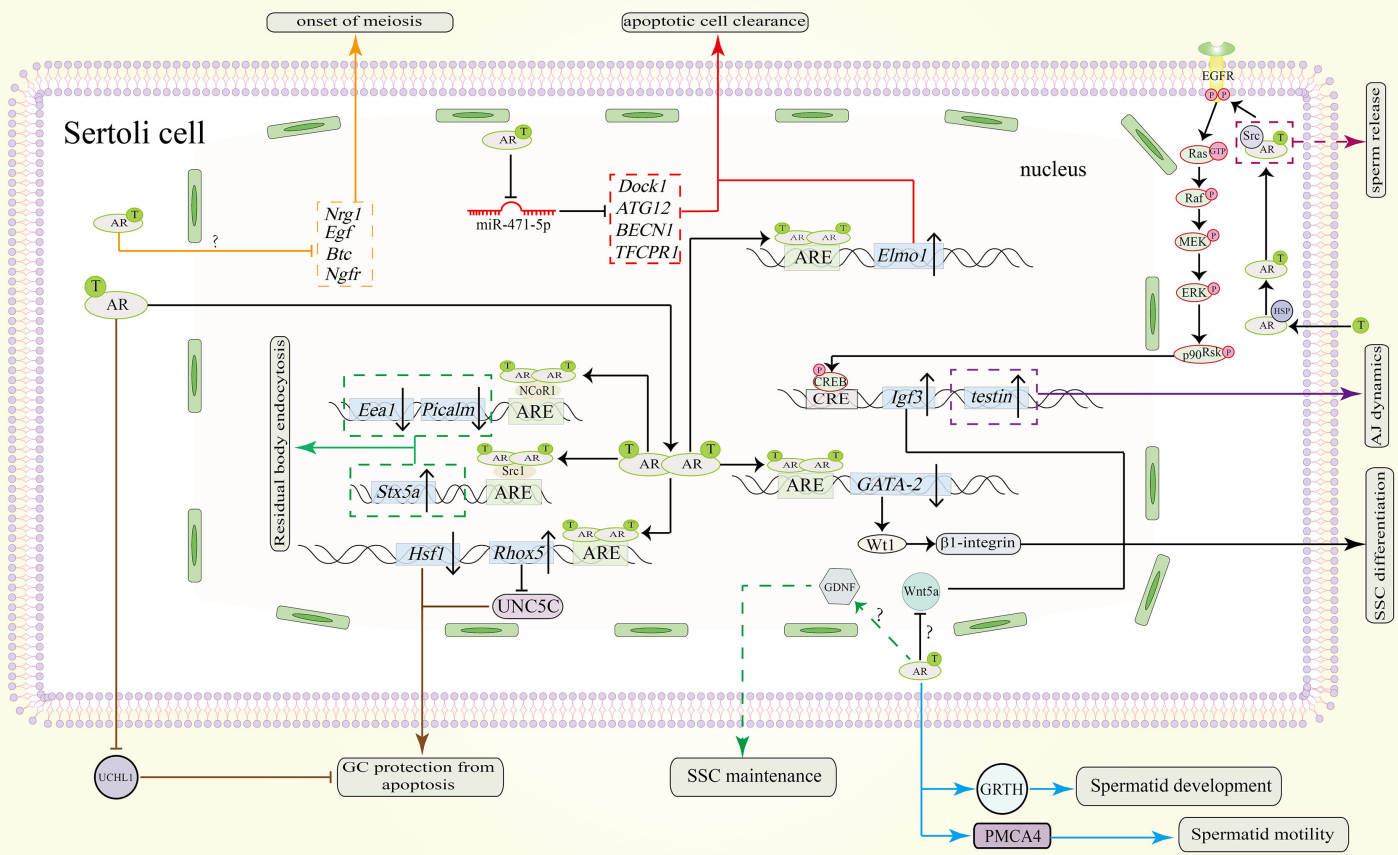
Function of the AR signaling pathway in SCs in spermatogenesis. AR dimers in the nucleus can bind to the ARE of *GATA-2* to inhibit its expression. Downregulated protein levels of GATA-2 downregulate Wt1 and β1-integrin to promote SSC differentiation. The AR also promotes SSC differentiation by repressing Wnt5a. However, the precise pathway between AR and Wnt5a remains unknown. Through unknown pathway, AR allows SCs to secret GDNF for SSC maintenance (shown in green dotted line). Testosterone can also induce the expression of *Igf3* through a non-classical signaling pathway to promote SSC differentiation. Through an unknown pathway, the AR represses the expression of *Nrg1*, *Egf*, *Btc* and *Ngfr* to induce the onset of meiosis (shown in orange). To protect GCs from apoptosis during meiosis, AR dimers bind to the AREs in the promoters of *Rhox5* and *Hsf1*. Upregulated protein levels of Rhox5 repress Unc5c expression (shown in brown). Downregulation of *Hsf1* can also protect GCs from apoptosis (shown in brown). Also, AR signaling in SCs can inhibit UCHL1 expression in spermatocytes to prevent them from apoptosis (shown in brown). In addition, the clearance of apoptotic cells is also important. Testosterone inhibits miR-471-5p and thereby upregulates the expression of *Dock1*, *ATG12*, *BECN1* and *TFCPR1*, resulting in clearance by SCs (shown in red). Elmo1 upregulation by AR dimerization can also promote this process (shown in red). During spermiogenesis and spermiation, non-classical signaling induces the expression of *testin*, which is needed for proper adherence junctions (AJ) dynamics (purple solid line). The residual body from the maturing sperm will be absorbed by the concerted action of Stx5a, Picalm and Eea1 (shown in green). AR dimers also bind to the coactivator Src1 to induce the expression of *Stx5a* and to the corepressor NCoR1 to repress the expression of *Picalm* and *Eea1*. Spermatid development is also regulated by AR signaling. AR signaling in SCs induce GRTH and PMCA4 transcription and expression in spermatids which will promote their development and motility respectively (shown in blue line). Sperm release is facilitated by Src-mediated molecular processes (indicated by the purple dotted line). ‘?’ indicates an unknown pathway.

Following the migration of gonocytes to the basement membrane and their differentiation into spermatogonia stem cells (5), SSCs are influenced by a network of signaling that can trigger SSC self-renewal and differentiation. AR signaling in SCs has been found to participate in these two processes, probably promoting SSC differentiation and repressing SSC self-renewal, although it is not clear whether both of classical and non-classical signaling in SCs contribute to these processes in all species (**Figure 3**).

#### 4.2.1 Wnt5a

Wnt5a, which refers to Wingless-type MMTV Integration Site Family Member 5A, is secreted by SCs in mice. Several studies found that *Wnt5a* expression is regulated by androgen and that it promotes SSCs self-renewal. Both *in vitro* and *in vivo* studies confirmed that Wnt5a can increase SSC self-renewal division after androgen blockage in mice SCs (84). In addition, Crespo et al. found that in zebrafish one androgen, 11-ketotestosterone (11-KT), indirectly inhibits SSC self-renewal by inhibiting prostaglandin E2 (PGE2) in SCs. Otherwise, PGE2 promotes SSC self-renewal by upregulating *Wnt5a* expression (85). However, more investigations should focus on the existence of AREs within the promoter of *Wnt5a* to confirm the exact signaling pathway in SCs through which androgen exerts its effects.

#### 4.2.2 Plzf

Promyelocytic leukemia zinc finger (*Plzf*), a key transcription suppressor gene, has been characterized as a marker for undifferentiated SSCs in rodents (86) and primates (87). *Plzf* is important for SSC maintenance (45). Recently, *in vivo* and *in vitro* studies found that after activation of AR signaling in SCs, AR homodimer binds to the ARE region of GATA binding protein 2 (*GATA-2*) to inhibit its expression; this can further decrease WT1 transcription factor (*Wt1*) expression since Wt1 expression requires binding of GATA-2to its promoter. Wt1 binds to β1-integrin and increases its expression in SCs; β1-integrin will interact with unknown molecules on the surface of SSCs, to induce *Plzf* expression. As a result, activation of classical AR signaling pathway can promote SSC differentiation by downregulating *Plzf* expression. We propose that the exact molecules that interact with β1-integrin on SSC surface should be identified to determine a more concise relationship between SCs and SSCs (88).

#### 4.2.3 Other Factors

In zebrafish, Insulin like growth factor 3 (*Igf3*) mRNA transcript level was found to be upregulated by androgen; this can promote SSC differentiation through non-classical signaling pathway (89). Reproductive homeobox 5 gene (*Rhox5)*, which is directly regulated by classical AR pathway, also contributes to spermatogonia differentiation (90). Besides, *in vitro* study found that testosterone can increase Glial-derived neurotrophic factor (GDNF) secretion by Sertoli cell (91). Glial-derived neurotrophic factor secreted will promote Gdnf family receptor α1 and proto-oncogene Rearranged expression during Transfection expression for SSC self-renewal and maintenance (92).

### 4.3 Role of AR Mediated Signaling in Spermatocytes Meiosis

In diploid organisms, the sexual differentiation requires the production of haploid gametes *via* meiosis, a process in which two consecutive cell divisions occur after a round of DNA replication (93). During prophase I of meiosis, leptotene and zygotene stages produce the DNA double strand breaks (DSBs) needed for DNA recombination, along with the chiasmata required for homologous chromosome separation. Following the zygotene stage is pachytene stage, in which the synapsis is completed, and DNA combination is finished as well as DSBs repair. In SCs, the AR signaling pathway is believed to be important in these stages (94) (**Figure 3**).

Loss of spermatocytes and spermatids in rodents occurred as a result of decreased intratesticular testosterone level in hypophysectomy, gonadotropin-releasing hormone antagonist treatments and hypogonadal (*hpg*) models (95). Above results indicate that testosterone mediated AR signaling is necessary for normal meiosis. Furthermore, inhibition of AR-Src interaction resulted in inactivation of non-classical signaling pathway in mice. Arrested meiosis was observed at the first spermatogenic wave (45). Moreover, in SCARKO mice where classical signaling pathway is blocked, De et al. observed loss of primary spermatocytes during stages VI-VII, a time when AR shows high protein expression level (96). These results suggest that both classical and non-classical AR signaling in SCs are indispensable for normal meiosis (**Figure 3**). In their attempt to determine the precise process that is regulated by AR signaling, Su et al. found that in SCARKO mice, meiosis arrest occurred in prophase I; cells failed to divide, and spermatocytes failed to acquire competence for the meiotic division phase (97). Although meiosis appeared to progress to a mid-pachytene-like cytological state, most germ cells stalled at an early-pachytene transcriptome stage (98). Precisely, meiosis arrest occurred between leptotene/zygotene to early–mid pachytene transition.

We conclude that there are two causes of meiotic arrest: chromosomal dysfunction and germ cell apoptosis.

#### 4.3.1 Chromosomal Dysfunction

The fact that AR expression peaks at stages VI-VIII of meiosis, coinciding with the preleptotene and leptotene stages, suggests that the AR might mediate the preparation of SCs for chromosomal synapsis and recombination. Male germ cells can enter prophase I of meiosis normally and undergo normal DSBs formation in the absence of AR signaling (5). However, the problems happened on DSBs repair and chromosome synapsis. In SCARKO mice, chromosome synapsis was incomplete, as shown by the fact that Chen et al. detected a significantly larger number of univalent chromosomes in spermatocytes than in the control group. Additionally, RAD51 and DMC1 which are required for resolution of DSBs and homologous recombination, along with TEX15, BRCA1, BRCA2 and PALB2 which load RAD51 and DMC1 onto DSBs sites, showed decreased protein expression in SCARKO mice. Furthermore, activation of AR can negatively regulate Epidermal growth factors (EGFs), including *Egf*, betacellulin (*Btc*) and neuregulin 1 (*Nrg1*). These EGFs bind to their corresponding receptors (EGFR, ERBB4) on the surface of spermatocytes and stimulate the accumulation of above homologous recombination factors for appropriate prophase preparation (94). At the synaptonemal complex, nuclear autoantigenic sperm protein (NASP) inhibits formation of CDC2/cyclinB1 complex *via* HSP70-2 which are important for G2/M transition. NASP showed elevated expression after androgen manipulation. Other genes that show changes in transcript after androgen manipulation, such as *Mapre1*, *Ruvbl1*, *Tuba1c*, *Tuba3a* and *Tubb2c*, are related to spindle dynamics and the formation of dynactin complexes (99). Although fewer spermatocytes may gain competence to enter metaphase I, they cannot complete meiosis due to the lack of normal spindle dynamics, and this may account for the disruption in post-meiotic differentiation. More proteins account for chromosome synapsis and dynamics that is under AR regulation deserve to be confirmed to establish the AR downstream signaling network by taking advantage of the development of proteomics.

#### 4.3.2 Germ Cell Apoptosis

GC apoptosis, especially apoptosis of spermatocytes, is another cause of meiotic arrest. This is not difficult to understand, since spermatocytes are cells that undergo meiosis. Through single cell transcript analysis technology, genes related to apoptosis have been identified. *Aldh1a1*, *Igfbps*, and mitochondrial membrane and oxidative phosphorylation transcripts were upregulated in SCARKO mice (98). Another group found that androgen deprivation led to a decreased expression of *Aldh2*, *Prdx6* and *Gstm5*, which encode protectors of oxidative stress (99). Other genes related to androgen-dependent apoptosis, such as *Diablo*, *Cbl*, *Hsf1* showed increased expression in mice after androgen deprivation or knocking out AR in SCs (97, 100). Hu et al. showed that Rhox5 can repress expression of Unc-5 Netrin Receptor C(UNC5C) which can promote spermatocyte apoptosis *in vitro* (101). Since Rhox5 is regulated by classical signaling pathway (90, 102, 103), it needs to be determined whether AR signaling represses UNC5C expression and thereby protects spermatocytes from apoptosis. Recently, Marina et al. found that ubiquitin carboxyl-terminal hydrolase L1(UCHL1) is negatively regulated by androgen. Decreased level of androgen leads to increased level of UCHL1 in spermatocytes, which will lead to deubiquitination of the pro-apoptotic factor p53. Deubiquitination of p53 triggers germ cell death (104). In addition, phagocytic clearance of apoptotic germ cells by SCs is vital for meiosis because phagocytosis of dying GCs provides lipid sources for SCs which will provide ATP for GCs development. Lower androgen increases the expression of miR-471-5p, resulting in inhibition of *Dock1, Tecpr1, Atg12* and *Becn1*. This leads to impaired LC3-associated phagocytosis. Besides, inhibition of *Dock1, Rac1-GTPase* in this model leads to disrupted engulfment of SCs (11). Both of these inhibitions impair clearance of apoptotic GCs by SCs. Another protein Elmo1, which is upregulated by classical AR signaling *in vivo*, participates in apoptotic germ cells clearance by SCs and functions downstream of Dock1 and Rac1-GTPase (73, 105).

Many of the transcription factors and regulators related to AR signaling that have been found are not mentioned here. These factors can participate in the regulation of meiosis. For additional information on these factors, please see **Table 1**. For additional genes that show changes in expression after androgen manipulation, please see the supplied references (98, 99, 123).

**Table 1:** Transcription regulators, upstream regulators and downstream regulators of Androgen receptor signaling during spermatogenesis.

Regulators			species	function		reference
Coactivator						
		ARID4B/4A	mice	BTB integrity		
				Timely SC maturation		(106, 107)
				Normal meiosis		
				Normal Rhox5 function		
				Post-meiotic differentiation		
		ARIP4	rats	Normal Rhox5 function		(108)
				Germ cells proliferation		
		SRC-2	human	Sustainable production of spermatozoa		(109)
		TRAM-1	human	Regulation of transcription		(110)
		PSPC1	mice	Promote AR transactivation		(111)
		NONO	mice	Promote AR transactivation		(111)
		SFPQ	mice	Promote AR transactivation		(111)
		SRC-1	rats	Promote residual body absorption		(112)
Corepressor						
		DjA1	mice	Inhibit spermatocyte death		(113)
				Promote round spermatid differentiation		
				Maintain adhesion junctions		
		HBO-1	human	Participate DNA replication		(109)
				Initiate and maintain spermatogenesis		
		NCOR1	rats	Promote residual body absorption		(112)
Upstream regulators						
	P110βPI3-kinase		mice		Spermatocyte differentiation	(114)
	Tzfp		mice		Normal cross-over through pachynema	(115)
					Germ cell formation	
					Repress AR signaling	
	NF-κB		rats		Activate transcription of AR	(116)
	LncNONO-AS		goats		Activate AR expression *via* NONO	(117)
Downstream regulators						
	Rhox5		mice		Enter prophase during prepuberty	(101, 102)
					Facilitate the first step of meiosis during puberty	
					Sperm release	
					BTB remodeling	
	FGF2		mice		Trigger spermatogonia proliferation and differentiation	(118)
					Meiosis initiation	
	Aard		mice		Normal spermatogenesis	(119)
					Regulate transcription activity	
	Cbl		rats		Activate androgen-dependent	(120)
	PMCA4		mice		Movement and motility of sperm	(121)
	Hsf1		mice		Protect immature gem cells	(100)
	Ube2b		mice		glycosylphosphatidylinositol (GPI)-anchor biosynthesis and oxidative phosphorylation	(82)
	Elmo1		mice		Sertoli cell mediated phagocytic clearance of apoptotic germ cells	(105)
	Spinlw1		rats		Sperm motility	(122)
	testin		mice		Adhesion junction dynamics	(113)

Based on the evidences presented above, we propose the reasons for meiotic arrest when AR signaling in SC is impaired. Chromosomal dysfunction or breakage can induce oxidative stress that cannot be repressed because of the decreased level of oxidative stress protectors. This leads to apoptosis of some spermatocytes. The presence of increased number of apoptotic cells, the failure to clear apoptotic cells combined with DNA damage trigger the inner meiotic checkpoint and cause meiosis to halt in prophase I. Future studies might focus on the roles of genes related to AR signaling that have been mined through mRNA transcript analysis in meiosis and attempt to determine whether they play permissive roles or instructive roles for meiosis.

### 4.4 Role of AR Mediated Signaling in Maintenance of Blood-Testis Barrier

SCs in mammalian testis can divide the seminiferous epithelium into basal and adluminal compartments through the construction of BTB. During stages VII-VIII of seminiferous epithelium cycle when the AR expression level peaks in rodents, BTB undergoes restructuring to facilitate the translocation of preleptotene spermatocytes from the basal area to the apical area where they will complete the entire processes of meiosis and post-meiotic processes. This transition, facilitated by testosterone, requires breakage of the ‘old’ BTB before preleptotene spermatocytes and formation of ‘new’ BTB after preleptotene spermatocytes entering into adluminal compartment (97, 124). Moreover, BTB shields haploid germ cells from recognition by the innate immune system; and this is why we also call it ‘immune barrier’ (125). BTB includes many types of junctions: tight junctions (TJs), gap junctions (GJs), desmosomes, basal ectoplasmic specialization (ES). These junctions undergo dynamic changes to fulfill their roles. Exposure of SC cultures to adenovirus constructs expressing inhibitors of either classical or non-classical AR signaling pathway led to increased permeability of BTB, suggesting that both classical and non-classical signaling pathway are important for BTB integrity, probably through regulating junction proteins (45, 126). (**Figure 2**).

#### 4.4.1 Tight Junction

Classical signaling pathway regulates proper tight junction dynamic. In mouse models with mutations in exon1 of AR, increased permeability of BTB was observed (124, 127). Although the BTB appears to be present in this model under microscopic examination, it is incomplete as we found biotin enter into adluminal area with the help of biotin tracer (127). Reversibly, BTB were strengthened after testosterone treatment of an SC culture system with disrupted AR function (128). To further investigate the molecular basis of these effects, microarray analysis was used to identify the genes that may be involved. The mRNA transcript level of tight junction protein including Claudin 3, Claudin 11, Claudin 13 were all downregulated while Occludin and Claudin 26 showed elevated transcript levels after ablation of exon1 of AR in mice (129). Among them, Claudin 3 which is not essential for fertility, is expressed in the newly formed BTB after preleptotene spermatocytes migrate past the old BTB (127). Claudin 13 shows a similar expression pattern to Claudin 3. CHIP-seq assays found putative AREs in the promoter regions of *Claudin 13* and *tight junction protein 2 isoform 3*, indicating a potential classical signaling regulation pattern. In contrast to Claudin 3, Claudin 11 preserves its expression throughout the seminiferous cycle. Besides, membrane-associated GUK family of proteins including *tight junction protein 1*, *tight junction protein 2 isoform 1*, *tight junction protein 2 isoform 2*, and *tight junction protein 2 isoform 3*, can function as a bridge that links tight junction proteins to the cytoskeleton like actin filaments. Their mRNA transcripts were found to be downregulated in mouse SCs after ablation of AR (129–131). What’s more, it was found that tight junction proteins can enter the nuclei of SCs and regulate their own transcription (132), indicating possible tight junction proteins compensatory effects in Cldn3^-/-^ mutant mice.

Apart from its directly regulation of TJ proteins, non-classical signaling can regulate TJ proteins *via* tissue type plasminogen activator (tPA) during stages VII-VIII of spermatogenesis when BTB undergoes restructuring (133). We also know that tPA is involved in BTB degradation (134), indicating a potential role of tPA in BTB dynamics. Moreover, Dietze et al. and Bulldan et al. found that Claudin 1, Claudin 5 and Claudin 11 protein levels were upregulated by CREB protein (49, 135, 136). These results further support the idea that non-classical androgen signaling can maintain BTB integrity by regulating tight junction dynamics.

Besides, in heat-treated monkey testes, AR regulates reversible changes in BTB integrity *via* Par complex (Par6-Par3, Par6-aPKC, and Par6-Cdc42) targeting TJ proteins (ZO-1 and occludin) and basal ES proteins (N-cadherin, E-cadherin, ɑ-catenin, β-catenin, and γ-catenin) (137).

Based on the results described above, we hypothesize that although the transition of preleptotene spermatocytes into adluminal compartment is not blocked when AR signaling in SC is prevented, the amount of claudin available for remodeling BTB (Claudin 3, Claudin 11, Claudin 13) is insufficient under these conditions. Besides, the downregulated tight junction proteins are not sufficient to enter the nucleus and increase their own transcription, let alone to recruit more claudins to the BTB. While Occludin and Claudin26 show relatively upregulated mRNA transcript levels, this is not enough to compensate for the loss of other tight junction proteins. Thus, humoral immunity against self-antigens is mounted, resulting in disruption of normal spermatogenesis.

#### 4.4.2 Gap Junction

Connexin 43 (Cx43), one gap junction protein, also under AR regulation as shown by its reduced mRNA transcript levels and protein expression levels in adult pig testes treated with flutamide, an anti-androgen (138). Interestingly, we also found weak AR immunostaining and partial disruption of AR signaling in SCs in Sertoli cell specific knock-out of connexin 43 mice (71). Xia et al. found that androgen can regulate the expression of Wt1 indirectly by inhibiting GATA-2expression. Androgen inhibits GATA-2 expression through the classical pathway. Wt1 can bind to the promoter of Cx43 and inhibit its expression, indicating that androgen can promote Cx43 expression by downregulating GATA-2 and Wt1 (139). What’s more, ouabain binding to its receptor ATP1A1can upregulate Cx43 expression *via* non-classical signaling pathway *in vivo* (140). The relationship between Cx43 and AR deserves further investigation. It remains to be determined whether reversing one of them (Cx43 or AR) can restore the other to normal level in human and correct impaired spermatogenesis, though it is possible that treating hpg mice with DHT can restore the expression and localization of Cx43 to the BTB (141).

#### 4.4.3 Blood-Testis Barrier Renewal

The endocytosis of old BTB proteins for renewal is also important for BTB integrity (142). Testosterone can promote the endocytosed occludin to the BTB location through recycling pathway (124). Other cells such as Madin-Darby canine kidney cells and epithelial cells can internalize occluding and claudin *via* clathrin- and caveolin-mediated pathways (143, 144). Coincidentally, Su et al. found that testosterone increases levels of clathrin and caveolin-1 in SCs (145), this suggests that AR signaling may participate in tight junction protein endocytosis for renewal *via* the clathrin and caveolin-1 pathways. This possibility needs further investigation.

Since AR signaling is important for BTB integrity, what will happen if AR is overexpressed under normal conditions? The influence of a tighter BTB on spermatogenesis remains unknown. We suggest an *in vitro* AR overexpression experiment to answer this question. Recently, in muscle cells, it was found that AR can participate in IGF-1/IGF-1R-PI3K/Akt-mTOR pathway (146). We also know that mTOR signaling can regulate BTB dynamic by balancing the levels of F-actin binding proteins epidermal growth factor receptor pathway substrate 8 and actin related protein 3 (147). Therefore, we suggest investigation into crosstalk between AR and mTOR pathway and its downstream molecules related to actin-binding proteins in germ cell lines.

### 4.5 Role of AR Mediated Signaling in Sertoli Cell - Spermatid Adhesion and Sperm Release

Before spermiogenesis occurs, round spermatids are linked to SCs by desmosomes. When the cycle of seminiferous epithelium enters into stage VII, the round spermatids begin to elongate accompanied by the replacement of desmosome anchors with new specialized adhering proteins. We call the structure formed in this way as apical ectoplasmic specialization (ES). The proteins that form ES disassemble before sperm release during stage VIII. The complexes connecting SC and elongated spermatids are tubulobulbar complex and the focal adhesion-related disengagement complex. AR signaling pathway is important in two processes during spermiogenesis and final spermiation: Sertoli cell – Spermatid adhesion and sperm release (**Figures 2**, **3**).

The importance of AR signaling in Sertoli cell - Spermatid adhesion and sperm release is elucidated by two *in vivo* model. First, in rats that received subdermal testosterone and oestradiol (TE) implants to lower the intratesticular testosterone level, researchers found loss of stage VIII and late spermatids (148, 149). Additionally, hypomorphic SCARKO mice displayed premature release of round spermatids as well as blockage of terminal differentiation and release of elongated spermatids. The unreleased mature spermatids were degenerated or phagocytized by SCs (149, 150).

#### 4.5.1 Sertoli Cell - Spermatid Adhesion

The molecular mechanism through which Sertoli cell - Spermatid adhesion occurs has been elucidated. Based on experiments in which adenovirus constructs were used to express different AR mutants that impair the classical or nonclassical pathways and on other experiments in which inhibitors of ERK kinase and Src kinase were added to an SC-GC coculture system, it is proposed by study that non-classical AR signaling pathway contributes dominantly to Sertoli cell-Spermatid adhesion, while activation of the classical AR signaling pathway is not sufficient to permit Spermatids binding to SCs (151). Testosterone can promote the attachment of spermatids to SCs in Sertoli cell -Spermatid co-culture system by activation ERK and Src, which belong to the non-classical signaling pathway (152). Terada et al. found that testin, which can be induced by non-classical signaling pathway, is important for Sertoli cell - Spermatid adhesion. Overexpression of testin can block spermatid differentiation, probably due to excessive adhesion to SCs (113). However, an *in vivo* study adopting TE implants brought about not only the loss of spermatids but also the activation of ERK before sperm release, along with breakage association of N-cadherin and β-catenin indicating disruption of ES (153). This result seems contradictory to *in vitro* study. One possible reason maybe that the formation of ES requires ERK activation only at the beginning of formation rather than during the entire process.

#### 4.5.2 Sperm Release

Round spermatids develop into elongated sperm before final releasing.

Gonadotropin Regulated Testicular Helicase (GRTH/DDX25) is significant in this process, mainly by participating on the nuclear export and transport of specific mRNAs as well as the structural integrity of Chromatoid Bodies of round spermatids (154). As an RNA helicase and only DEAD-box family member regulated by androgen in spermatids, GRTH/DDX25 transcription in spermatid is regulated by AR signaling pathway in Sertoli cells through a paracrine fashion. Germ cell nuclear factor (GCNF) in spermatids can promote GRTH/DDX25 transcription and expression in response to androgen regulation (155–158). However, the signal relayed to spermatids remains to be further elucidated.

Before releasing sperm, SCs endocytose the residual body from maturing sperm. Kumar et al. found that AR dimerization can recruit corepressor nuclear receptor corepressor 1 (NCoR1) to the ARE of phosphatidylinositol binding clathrin assembly protein (*Picalm)* and early endosome antigen 1 (*Eea1)* and thereby inhibit their expression. Coactivator steroid receptor coactivator-1 (Src1) can be recruited to ARE of syntaxin 5 (*Stx5a)* and induce its expression (112, 159, 160). Since the products of these 3 genes are located around tubule bulbar complexes and participate in endocytosis and intracellular transport, it is possible that they are involved in residual body absorption.

Activation of Src is important for sperm release (161, 162). The injection of Src inhibitor into rat testis can block the release of elongating spermatids, while injection of an ERK inhibitor has little effect on sperm release (152, 163). Additionally, O’Donnell et al. pointed out that the disengagement complex, including α6β1 integrin which associates with Src, and tyrosine-phosphorylated focal adhesion kinase (FAK), remains associated with sperm that fail to be released. Both Src and FAK can be phosphorylated on activation of non-classical androgen signaling (149). Further study confirmed that activated non-classical signaling target Src is near the head of sperm to be released (164).

Also, sperm motility is significant during sperm release. Located in elongated spermatids, ATPase Ca^++^ transporting plasma membrane 4 (PMCA4) is critical for sperm motility (165). Recently, it was found that PMCA4 is positively regulated by AR signaling in SCs both *in vitro* and *in vivo* (121).

In a summary, non-classical AR signaling promotes Sertoli cell – Spermatid adhesion by activating both ERK and Src while promoting sperm release by activating Src. Considering the above study, future studies might focus on achieving a deeper understanding of the different effects of Src to immature spermatid and mature spermatid as well as the impact of both ERK and Src to other kinds of proteins in apical ES.

## 5 Discussion on Androgen Insensitivity Syndrome: Present Advances and Future Therapies

Infertility is an emerging worldwide public health issue. Approximately 20-70% of cases are due to male infertility (166). For most men suffering from infertility, the primary cause is low quality and low quantity of sperm (167). Hormones, including androgens, are necessary for spermatogenesis. We have reviewed AR signaling in SCs above. Clinical mutations in androgen receptor can cause reproductive diseases such as Androgen insensitivity syndrome, Sertoli cell-only syndrome and Obstructive azoospermia. Here we will focus on androgen insensitivity syndrome, which is closely related to AR mutations in SCs.

Androgen insensitivity syndrome (AIS) is an X-linked recessive disorder of sex development in patients with the 46, XY karyotype (168). According to the National Institutes of health, approximately 2-5 in 100000 people may have AIS. The biochemical mechanism behind AIS is the inability of cells expressing AR to respond to androgen, leading to incomplete or absent genital virilization of the 46, XY embryo (169). Specifically, the mutations in AR result in disrupted capacity of binding to testosterone or DHT, leading to impaired differentiation of the Wolffian ducts and impaired virilization of the external male genitalia respectively (170). At present, AIS is classified into two categories, AIS type I and AIS type II. In AIS type I, mutations exist in AR itself; this type of AIS can be further classified into complete AIS (CAIS), partial AIS (PAIS) and mild AIS (MAIS) according to the AR mutation level, while mutations in AIS type II occur in molecules downstream of AR rather than in AR itself (171). The clinical phenotype of CAIS is characterized by an external female phenotype with undescended testes (172), PAIS leads to different ranges of hypospadias and ambiguous genitalia (173) and individuals with MAIS display a male phenotype, sometimes with gynecomastia, and insufficient sperm production (174). Recent years, studies and many case reports have revealed different AR mutations in patients with AIS. Mutations found up to now include gene deletion, mutations in transactivation domain, mutations in the DNA binding domain, mutations in the ligand binding domain. (For additional information, please see review (175)) In addition, even individuals without a mutation in the AR gene can show an AIS-like syndrome, indicating that mutations may have occurred somewhere else (176).

Currently, researches on AIS focus mainly on recording patient phenotypes and detecting AR mutations through sequencing of AR gene. Various AR mutations have been found to date (177). Inadequate research on associated gene expression patterns presents much difficulties for the development of therapies, especially for type II AIS. In addition, few studies have used testicular histology to examine the effects of AR mutations on testis. Recently, one CAIS patient showed unusually high level of AMH and SOX9, accompanied by immature SCs, a phenomenon that has been linked in a mouse model to the AR/SOX9/AMH pathway discussed above (178). AMH, along with Inhibin B, which may have positive or inverse correlation with AMH, is thought to be appealing subject for study by endocrinologists because patients with androgen insensitivity or defects in androgen synthesis have normal or high levels of AMH (179).

Notably, a high level of estrogen was found in patients with CAIS, and this may explain the development of breast cancer in some of these patients. Supporting this idea, it is known that testosterone can be converted into estrogen *via* aromatase and luteinizing hormone can stimulate testicular estrogen synthesis. Researchers have found high levels of luteinizing hormone and testosterone in AIS patients (168, 175, 180). Interestingly, apolipoprotein D (APOD), which is induced by DHT, is a potential candidate marker for the detection of AIS, especially for type II AIS. Treating type II AIS patients with DHT can induce endogenous APOD expression but the level of APOD is below the calculated cut-off (181). Other molecular pathways downstream of AR in AIS patients are remaining to be elucidated. One question arises for our molecular research. Although mice and rats have high homology with humans, some phenotypes in AIS patients cannot be reproduced in mouse or rat models. Although this is a significant barrier in our research, we should not be pessimistic. In a comparison of control males and CAIS females, significant differences in the transcription levels of 612 genes were found at the cellular level, suggesting that androgens may play a role in transcriptome regulation in humans (182). We are pleased to see that AR can regulate the transcriptome level in mouse models (98); this observation can guide us to investigate genes with known function in mouse models in our clinical diagnosis and to research genes that show no overlap between two species in mice to determine their function.

In summary, our opinions are that the diagnosis for AIS should combine power from different fields of study. For geneticists, family history is recommended to record for prenatal diagnosis, along with determining the patient’s karyotype after birth (183). Some important biomarkers, such as AMH, estrogen, luteinizing hormone and testosterone should be measured at birth or at puberty to distinguish AIS from androgen synthesis deficiency by endocrinologists. Cytologists and histologists use fine needle aspiration cytology (184) and histopathology to examine the state of SCs and the testicular lumen, and the results obtained may be compared with results obtained from patients with other syndromes like, such as Sertoli cell-only syndrome or nonobstructive azoospermia, possibly benefiting from therapies for those diseases. Finding associated genes and impaired signaling pathway are tasks for molecular biologists. Combined efforts are powerful.

At present, the most common treatment for CAIS is gonadectomy in early adulthood. There are two reasons for this: 1) an undescended testis has an increased risk of malignant transformation after puberty; and 2) testosterone produced by the testis can be converted to estrogen, so puberty will occur naturally (185, 186). Besides, treatment for PAIS vary between different patients. Treatments currently include androgen supplementation at puberty, surgeries to repair hypospadias and bring undescended testes into the scrotum. Personal treatment should combine diagnosis, sex assignment and biochemical examination (187). Previous study using AR transgene model restored the impaired spermatogenesis in SC AR mutant mice (188). Whether transfection of AR constructs into human SCs can benefit AIS patients remains to be elucidated.

Moreover, the job of a doctor is not only to treat patients physically, but also to treat them psychologically. Helping AIS patients overcome their psychological disorders and live a normal life without discrimination is a responsible behavior for doctors and for society as a whole.

## 6 Conclusion and Perspective

Both classical and non-classical androgen receptor signaling pathway are essential for spermatogenesis. These two pathways not only regulate genomic action in Sertoli cells but also influence germ cells between Sertoli cells through paracrine action. Sertoli cells are expected to be a new therapeutic target. However, rare studies focus on the relationship between androgen receptor signaling pathway, Sertoli cell and clinical diseases. The transgenic model developed recently which is able to activate only classical or non-classical signaling pathway, allows us to screen more genes related to androgen receptor signaling pathway. These genes are helpful for us to develop therapies for treating infertility. Moreover, spermatogonia transplantation is a method to inject spermatogonia into seminiferous tubule lumen and promote spermatogonia to form colonization at basement area, which may cure fertility fundamentally. The problem about making spermatogonia migrating from adluminal area to basement area may be solved by androgen therapies because lowering testicular testosterone level can open BTB reversibly.

## Author Contributions

J-MW, Z-FL, and W-XY conceived of and authored the paper. All authors contributed to the article and approved the submitted version.

## Funding

This work was supported in part by National Natural Science Foundation of China (No 32072954).

## Conflict of Interest

The authors declare that the research was conducted in the absence of any commercial or financial relationships that could be construed as a potential conflict of interest.

## Publisher’s Note

All claims expressed in this article are solely those of the authors and do not necessarily represent those of their affiliated organizations, or those of the publisher, the editors and the reviewers. Any product that may be evaluated in this article, or claim that may be made by its manufacturer, is not guaranteed or endorsed by the publisher.

## Glossary

**Table:**

11-KT	11-ketotestosterone
Aard	alanine and arginine rich domain
AIS	Androgen insensitive syndrome
AMH	Anti Mullerian hormone
APOD	apolipoprotein D
ARE	Androgen response element
ARID4A/AB	AT-rich interaction domain 4A/4B
ARIP4	androgen receptor-interacting protein 4
AR	androgen receptor
ARKO	Androgen receptor knock out
BTB	Blood-testis barrier
Btc	betacellulin
CAIS	Complete androgen insensitive syndrome
Cbl	calcineurin B-like protein
CK18	Cytokeratin 18
CRE	cAMP-response element
CREB	cAMP-response element-binding protein
Cx43	connexin 43
DHT	Dihydrotestosterone
DjA1	type 1 DnaJ protein
DSBs	DNA double strand breaks
Eea1	early endosome antigen 1
EGFR	Epidermal growth factor receptor
Elmo1	engulfment and cell motility 1
ES	Ectoplasmic specialization
FAK	Focal adhesion kinase
FGF2	Fibroblast growth factor 2
GATA-2	GATA binding protein 2
GC	Germ cell
GCNF	Germ cell nuclear factor
GDNF	Glial-derived neurotrophic factor
GJ	gap junction
GPI	Glycosylphosphatidylinositol
GRTH/DDX25	Gonadotropin Regulated Testicular Helicase
hpg	hypogonadal
Igf3	Insulin like growth factor 3
Hsf1	Heat shock transcription factor1
lncNONO-AS	target NONO lncRNA
MAIS	Mild androgen insensitive syndrome
NASP	nuclear autoantigenic sperm protein
NCoR1	nuclear receptor corepressor 1
Nrg1	neuregulin 1
NONO	non-POU domain-containing octamer-binding protein
p^90RSK^	ribosomal protein S6 kinase A1
PAIS	Partial androgen insensitive syndrome
PGE2	prostaglandin E2
PI3K	phosphatidylinositol 3 kinase
Picalm	phosphatidylinositol binding clathrin assembly protein
Plzf	Promyelocytic leukemia zinc finger
PMCA4	ATPase Ca^++^ transporting plasma membrane 4
PSPC-1	paraspeckle component 1
Rhox5	Reproductive homeobox 5 gene
RNF4	really interesting new gene (RING) finger protein 4
SC	Sertoli cell
SCARKO	Sertoli cell androgen receptor knock out
SF1	steroidogenic factor 1
SFPQ	splicing factor proline/glutamine rich
SOX9	sex determining region Y-box 9
Spinlw1	Epididymal protease inhibitor
Src	SRC proto-oncogene
Src1	steroid receptor coactivator-1
SSC	Spermatogonia stem cell
Stx5a	syntaxin 5
T	testosterone
TE	testosterone and oestradiol
Tfm	testicular feminized mouse
tPA	type plasminogen activator
TJ	tight junction
TRAM-1	thyroid hormone receptor activator molecule-1
TUBB3	Class III βtubulin
Tzfp	testis zinc finger protein
Ube2b	ubiquitin-conjugating enzyme E2B
UCHC1	ubiquitincarboxyl-terminal hydrolase L1
UNC5C	Unc-5 Netrin Receptor C(UNC5C)
Wnt5a	Wingless-type MMTV Integration Site Family Member 5A
Wt1	WT1 transcription factor
ZIP9	Zrt- and Irt-like protein 9.
